# Intervention in Context-Sensitive Probabilistic Boolean Networks Revisited

**DOI:** 10.1155/2009/360864

**Published:** 2009-02-11

**Authors:** Babak Faryabi, Golnaz Vahedi, Jean-Francois Chamberland, Aniruddha Datta, EdwardR Dougherty

**Affiliations:** 1 Department of Electrical and Computer Engineering, Texas A&M University, College Station, TX 77843, USA; 2 Computational Biology Division, Translational Genomics Research Institute, Phoenix, AZ 85004, USA

## Abstract

An approximate representation for the state space of a context-sensitive probabilistic Boolean network has previously been proposed and utilized to devise therapeutic intervention strategies. Whereas the full state of a context-sensitive probabilistic Boolean network is specified by an ordered pair composed of a network context and a gene-activity profile, this approximate representation collapses the state space onto the gene-activity profiles alone. This reduction yields an approximate transition probability matrix, absent of context, for the Markov chain associated with the context-sensitive probabilistic Boolean network. As with many approximation methods, a price must be paid for using a reduced model representation, namely, some loss of optimality relative to using the full state space. This paper examines the effects on intervention performance caused by the reduction with respect to various values of the model parameters. This task is performed using a new derivation for the transition probability matrix of the context-sensitive probabilistic Boolean network. This expression of transition probability distributions is in concert with the original definition of context-sensitive probabilistic Boolean network. The performance of optimal and approximate therapeutic strategies is compared for both synthetic networks and a real case study. It is observed that the approximate representation describes the dynamics of the context-sensitive probabilistic Boolean network through the instantaneously random probabilistic Boolean network with similar parameters.

## 1. Introduction

In biology, there are numerous examples where the (in)activation of one gene or protein can lead to a certain cellular functional state or phenotype. For instance, in a stable cancer cell line, the reproductive cell cycle is repeated, and cancerous cells proliferate with time in the absence of intervention. One can use the p53 gene if the intervention goal is to push the cells into apoptosis, or programmed cell death, to arrest the cell cycle. The p53 gene is the most well-known tumor suppressor gene, encoding a protein that regulates the expression of several genes such as Bax and Fas/APO1, which function is to promote apoptosis [1, 2]. In cultured cells, extensive experimental results indicate that when p53 is activated, for example, in response to radiation, it leads to cell growth inhibition or cell death [3]. The p53 gene is also used in gene therapy, where the target gene (p53 in this case) is cloned into a viral vector. The modified virus serves as a vehicle to transport the p53 gene into tumor cells to generate intervention [4, 5]. As this and many other examples suggest, it is prudent to use gene regulatory models to design therapeutic interventions that expediently modify the cell's dynamics via external signals. These system-based intervention methods can be useful in identifying potential drug targets and discovering treatments to disrupt or mitigate the aberrant gene functions contributing to the pathology of a disease.

The main objective of intervention is to reduce the likelihood of encountering the undesirable gene-activity profiles associated with aberrant cellular functions. Probabilistic Boolean networks (PBNs), a class of discrete-time discrete-space Markovian gene regulatory networks, have been used to derive such therapeutic strategies [6]. These classes of models, which allow the incorporation of uncertainty into the inter-gene relationships, are probabilistic generalizations of the standard Boolean networks introduced by Kauffman [7–9]. In a PBN model, gene values are quantized into some finite range. The values are updated synchronously at each time step according to regulatory functions. Stochastic properties are introduced into the model by allowing several possible regulatory functions for each gene and allowing random modification of the activity factors. If the regulatory functions are allowed to change at every time point, then the PBN is said to be *instantaneously random* [10]. On the other hand, in a *context-sensitive* PBN, function updating only occurs at time points selected by a binary switching random process [11, 12]. This framework incorporates the effect of latent variables outside the model, whose behaviors influence regulation within the system. In essence, a PBN is composed of a collection of networks; between switches it acts like one of the constituent networks, each being referred to as a *context*. The switching frequency of the context differentiates the instantaneously random PBN from the context-sensitive PBN.

The context switching that occurs at every time step in an instantaneously random PBN corresponds to changing the wiring diagram of the system at every instant. In contrast, context-sensitive PBNs can better represent the stability of biological systems by capturing the period of sojourning in constituent networks [11]. Hence, this class of models is more suitable for the analysis of gene regulation and the design of intervention methods. To formulate the problem of intervention in a context-sensitive PBN, a transition probability matrix must be derived. This transition matrix acts on the possible states of the system. Once this is accomplished, the task of finding the most effective intervention strategy can be formulated as a classical sequential decision making problem. For a predefined cost of intervention and a cost-per-stage function that discriminates between the states of the system, the objective of the decision maker becomes minimizing the expected total cost associated with the progression of the system. That is, given the state of the system, an effective intervention strategy identifies which action to take so as to minimize the overall cost. Consequently, the devised intervention strategy can be used as a therapeutic strategy that alters the dynamics of aberrant cells to reduce the long-run likelihood of undesirable gene-activity profiles favorable to the disease. It is evident that the intervention strategy specified by the sequential decision maker is directly affected by the form of the transition probability matrix associated with a context-sensitive PBN. For an instantaneously random PBN, the state consists of a gene-activity profile; while for a context-sensitive PBN, the state includes a gene-activity profile and a context.

The effectiveness of an intervention strategy depends, partly, on how accurate the model represents the reality of the underlying pathological cellular functions. It is therefore important to adopt a model that captures the subtleties of the biological system of interest. In the framework of context-sensitive PBNs, this entails defining a transition probability matrix that is an accurate representation of system dynamics. Context-sensitive PBN models have been considered in [13, 14]. In [13], an intervention strategy is devised for a limited window of observations. Although this method lowers the likelihood of undesirable gene-activity profiles within the control window, it may not alter the probability of visiting these gene expression profiles in the long run. To address this issue, [14] derives a stationary strategy that affects the long-run behavior of gene-activity profiles.

Common to [13, 14] is the assumption that the active constituent network of the context-sensitive PBN is not observable at each instant. This means that decisions must be made without explicit knowledge of the context, and therefore without full knowledge of the system state, which is composed of a context and a gene-activity profile. As such, the authors of [13, 14] elect to proceed using a transition probability matrix in which the context is removed from the state space and system dynamics. This reduction is accomplished by computing a weighted sum of the gene-activity profile behaviors over all the possible constituent networks. At every step, the reduced system exhibits an expected behavior by averaging over the various contexts. As such, the gene-activity profile determines the status of the approximate system, and the collapsed transition probability matrix specifies its evolution. The corresponding intervention strategy is based on the approximate transition probability matrix with the collapsed state space. Not only does the reduction eliminate the need to know the context at each time point, but it also reduces the dimensionality of the control problem. For instance, in a binary context-sensitive PBN with  genes and  contexts, the full state space consists of  states, whereas the reduced system possesses  states. Consequently, the computational complexity of each iteration of the intervention design algorithm is  for the context-sensitive PBN, whereas it is  for the reduced system [15].

Although the reduction in [13, 14] has benefits, as with many approximation methods, a price must be paid, and here it arises from the intervention standpoint. Specifically, what is the cost in terms of the effectiveness of the resulting intervention strategy? This issue is not addressed in [13, 14]. Our aim here is to determine, under various network parametric assumptions, the loss of intervention performance resulting from removing the context from the state space of a context-sensitive PBN. This must be done by comparing the performance of the intervention strategies derived from the full state space and the reduced state space when both are individually applied to the full state space. In [13, 14], the strategy devised on the reduced space was never actually applied to the original system. It was only applied to the approximate model. The point is that the performance of the approximate strategy was tested on the reduced model, not on the original one. As such the cost of the reduction was never assessed. This is accomplished below. This approximation simplifies the task of finding intervention strategies by describing the dynamics of a context-sensitive PBN via the instantaneously random PBN with similar parameters, and hence it should be expected to be accurate mostly when contexts switch frequently.

In Section 2, we review the definition of a context-sensitive PBN. We briefly explain a method to design strategies for controlling the dynamical behavior of the model in the long run using classical Markov decision processes. A new derivation for the transition probability matrix of a context-sensitive PBN is presented in Section2.3. In Section 2.4, we review the reduction method proposed in [13, 14] and derive the corresponding approximate transition probability matrix using the results of Section 2.3. We compare the performance of approximate and optimal intervention strategies through extensive numerical studies in Section 3. In this section, we also formulate a seven-gene context-sensitive PBN model for a melanoma case study [16]. The performance of the optimal and approximate intervention strategies for this network is compared under various model parameters.

## 2. Intervention in Context-Sensitive Probabilistic Boolean Networks

We begin this section with a review of context-sensitive PBNs and then formulate the problem of intervention as an infinite-horizon sequential decision making problem. We derive a new expression for the transition probability matrix that specifies the dynamics of the system based on its regulatory functions. This expression for the transition probability matrix is in concert with the original definition of context-sensitive PBNs [12]. As noted previously, the state space of the associated transition probability matrix is composed of all possible context and gene-activity profile pairs. We conclude this section by presenting an approximate transition probability matrix derived by performing a state collapse over the various contexts. This approximation method was used in [13, 14]. Mathematically, this is a Markov approximation to a hidden-Markov model. In the approximate transition probability matrix, the probability of moving from one gene-activity profile to another is the weighted sum of the probability transitions between these two states under the various contexts. The coefficients of the weighted summation are the selection probabilities of the contexts.

### 2.1. Definition

A context-sensitive probabilistic Boolean network consists of a sequence  of  nodes, where , and a sequence  of vector-valued functions defining constituent networks. In the framework of gene regulation, each element  represents the expression value of a gene. It is common to abuse terminology by referring to  as the th gene. Each vector-valued function  represents a constituent network of the context-sensitive PBN. The function  is the predictor of gene , whenever context  is selected. The number of quantization levels for gene expressions is denoted by . At each updating epoch, a random variable determines whether the constituent network is switched or not. The switching probability  is a system parameter. If the context remains unchanged, then the context-sensitive PBN behaves like a fixed Boolean network where the values of all the genes are updated synchronously according to the current constituent network. On the other hand, if a switch occurs, then a constituent network is randomly selected from  according to the selection probability distribution . Once the predictor function  is determined, the values of the genes are updated using the new constituent network, that is, according to the rules defined by .

Two quantization levels have thus far been used in practice. If  (binary), then the constituent networks are Boolean networks with  or  meaning OFF or ON, respectively [10]. The case where  (ternary) arises when we consider individual genes to be downregulated (0), upregulated (2), or invariant (1). This situation commonly occurs with cDNA microarrays, where a ratio is taken between the expression values on the test channel (red) and the base channel (green) [16]. In this paper, we will develop the methodology for , so that gene values are either  or . The methodology can be extended to other finite quantization levels, albeit, at the expense of tedious mathematical expressions. All the binary operations in this section would need to be replaced by case statements, and the perturbation process should be articulated on a case by case basis.

We focus on context-sensitive PBNs with perturbations, meaning that each gene may change its value with small probability  at each epoch. If  is a Bernoulli random variable with parameter  and the random vector  at instant  is defined as , then the value of gene  is determined at each epoch  by(1)

where operator  is componentwise addition in modulo two and  is the predictor of gene  according to the current context of the network . Such a perturbation captures the realistic situation where the activity of a gene is subject to random alteration.

The gene-activity profile (or GAP) is an -digit binary vector  giving the expression values of the genes at time , where . We denote the set of all possible GAPs by . The dynamic behavior of a context-sensitive PBN can be modeled by a Markov chain whose states are ordered pairs consisting of a constituent network  and a GAP . The evolution of the context-sensitive PBN can therefore be represented using a stationary discrete-time equation(2)

where state  is an element of the state space . The disturbance  is the manifestation of uncertainties in the biological system, due either to context switching or a change in the GAP resulting from a random gene perturbation. It is assumed that both the gene perturbation distribution and the network switching distribution are independent and identically distributed over time. The switching frequency of the context differentiates the instantaneously random PBN from the context-sensitive PBN. If the contexts change at every instant, that is, , then the PBN is instantaneously random.

We note that a bijection can be drawn between the gene-activity profile  or the states  and their decimal representations  and  based on their binary expansion. The integers  and  take values in  and , respectively. These decimal representations facilitate the depiction of our numerical results in Section 3.

### 2.2. Infinite-Horizon Intervention

We can formulate the task of finding the most effective intervention strategy as a sequential decision making problem, when the dynamics of a context-sensitive PBN are expressed according to (2). To this end, we can specify the Markov chain that describes the dynamics of the context-sensitive PBN by defining its transition probability matrix and initial state distribution.

In the presence of an external regulator, we suppose that the context-sensitive PBN has a binary intervention input  on the control gene . The intervention input , which takes values in set , specifies the action on the control gene . Treatment alters the status of the control gene , which can be selected from all the genes in the network. If treatment is applied, , then the state of the control gene  is toggled; otherwise the state of the control gene  remains unchanged.

For the case of a single control gene , the system evolution is represented by a stationary discrete-time equation(3)

where the state  is an element of , and similar to the context-sensitive PBN without control,  is the manifestation of uncertainties in the model. The transition probability matrix for the controlled context-sensitive PBN can be defined easily, once the transition probability matrix of the uncontrolled system is known. We derive an expression for this matrix in Section2.3. Originating from a state , the successor state  is selected randomly within set  according to the transition probability ;(4)

for all  and all .

To define the problem of intervention in a context-sensitive PBN, we associate a cost-per-stage  to each possible event. In general, the cost-per-stage can depend on the origin state , the successor state , and the control input . We define the average immediate cost in state , when control  is selected, by(5)

We consider a discounted formulation of the expected total cost. The discounting factor, , ensures convergence of the expected total cost over the long run [17]. In the case of cancer therapy, the discounting factor also emphasizes that obtaining treatment at an earlier stage is favored over later stages.

For initial state  and strategy , where  denotes the decision rule at epoch , the infinite expected total discounted cost is defined by(6)

The sequential decision maker must identify an optimal strategy  such that the  is minimized for each state . Mathematically, an optimal strategy  is a solution of the optimization problem(7)

For the specifics of our formulation, an optimal strategy always exists [17]. It is given by the fixed-point solution of the Bellman optimality equation(8)

Moreover, this optimal strategy is stationary and independent of the initial state  [17]. Standard dynamic programming algorithms can be used to find a fixed-point of the Bellman optimality equation. In our model, gene perturbation ensures that all the states communicate with one another. Hence, the Markov decision process associated with any stationary policy is ergodic and has a unique invariant distribution equal to its limiting distribution [18].

### 2.3. Transition Probability Matrix of a Context-Sensitive Probabilistic Boolean Network

For a given cost of intervention and cost-per-stage, the solution to (8) depends on the transition probability matrix in (4). The latter can be found by observing that two mutually exclusive events may occur at any epoch: the current context of the network remains the same for two consecutive instants, or the context of the network changes to a new one at the time instant . Moreover, the context may remain unchanged in two mutually exclusive ways: the binary switching variable is , which means that no change is possible, or the binary switching variable is , and the current network is picked again through random selection [11, 12]. In particular, when the switching variable is , a new context is selected independent of the current system state. Thus, the same network can be active twice in a row. This interpretation of switching the context in a PBN is in concert with the original definition of context-sensitive PBNs in [12]. Before proceeding, we note that transitioning was defined differently in [13, 14], where it was assumed that, whenever the switching variable is , a change of context must occur; the result being that context selection is conditioned on the current context. While this contrast produces a difference in the transition probabilities, it does not change the underlying issue in this paper, that being analyzing the effects of the state-space reduction proposed in [13, 14] on the performance of therapeutic interventions.

Letting  and  be two states in , we derive the transition probability(9)

from  to  in the absence of intervention. Note that we can rewrite expression (9) as(10)

Using the Bayes theorem, we get(11)

where  is the indicator function. Furthermore, we have(12)

and when , we get(13)

Here,  is the probability of switching context, and  is the probability of selecting context  during a switch.

A transition from GAP  to GAP  may occur either according to the constituent network at instant  or through an appropriate number of random perturbations, but not both. Let us define  by(14)

Then, we have(15)

and, for , we obtain(16)

where  is the Hamming distance between two gene-activity profiles  and .

The first parts of (15) and (16) correspond to the probability of transition from GAP  to GAP  according to the predictor functions defined by the constituent network at time instant . The remaining terms account for transition between GAPs that are due to random gene perturbation.

By replacing the terms of expression (11) by their equivalents from (12), (13), (15), and (16), it can be shown that the probability of transition from any state  to  is given by(17)

The elements of the transition probability matrix of the controlled context-sensitive PBN given by (4) can then be expressed through (17). The value of state after intervention  can be determined according to the status of the control signal and the value of the state prior to the intervention . Here,  is equated to , and the value of the GAP is updated according to the value of the control signal in the devised strategy according to(18)

All the  elements of vector  are zeros except the element at the th position, which is set to one.

### 2.4. Approximate Transition Probability Matrix of a Context-Sensitive Probabilistic Boolean Network

Following the reduction method proposed in [13, 14], we derive an expression for the approximate transition probability matrix in which the context is removed from the state space of the system. We base our derivation on the stochastic matrix defined by (17). The approximate stochastic model describes the dynamics of the system solely based on the GAPs, and its state space takes values from the set . The probability of transition from GAP  to GAP  in two consecutive epochs is derived from the weighted sum of the actual transition probabilities with respect to the selection probabilities of the contexts. If we denote the probability of transition between two GAPs by(19)

then under the reduction assumptions, we define(20)

Moreover, we can expand this expression as(21)

which in turn can be presented as(22)

where(23)

The above expression for  can be further simplified as(24)

Thus, we have(25)

The last expression for  can be further reduced to(26)

by setting .

Equations (22) and (26) express the approximate transition probability matrix associated with the reduced model. Although we have started from a different expression for the transition probability matrix of a context-sensitive PBN owing to a different interpretation of switching contexts, our final expression for the approximate transition probability matrix is similar to the one in [13, 14]. Averaging over the various contexts in (20) reduces the transition probability distributions associated with a context-sensitive PBN to transition probability distributions arising from the corresponding instantaneously random PBN, the fact that is overlooked in [13, 14]. The transition probability matrix of the corresponding instantaneously random PBN with the similar parameters can be obtained from expression (17), when the context is allowed to switch at each epoch by setting . Hence, the optimal and approximate intervention strategies perform similarly whenever the switching probability approaches to value one. This observation is supported by our numerical studies.

## 3. Numerical Results

In this section, we compare the performance of algorithms based on the exact and approximate expressions for the transition probability matrix associated with a context-sensitive PBN. We perform this comparison first through extensive simulations based on randomly generated context-sensitive PBNs. We then compare these methods for a network obtained from a melanoma gene-expression data set, which is similar to the one used in [13, 14].

### 3.1. Synthetic Networks

In our numerical studies, we postulate the following cost-per-stage:(27)

where  is the cost of control, and ,  are the sets of undesirable and desirable states, respectively. We set  to make the application of intervention more plausible compared to visiting undesirable states. The target gene is chosen to be the most significant gene in the GAP. We assume that the upregulation of the target gene is undesirable. Consequently, the state space is partitioned into desirable states, , and undesirable states, , where  is the number of genes. We use the natural decimal bijection of the GAP  to facilitate the presentation of our results. We set the number of genes to be five. The study of networks with larger numbers of genes would be computationally prohibitive due to the complexity of the corresponding dynamic program. The cost values have been chosen in accordance with an earlier study [14]. Since our objective is to downregulate the target gene, a higher cost is assigned to destination states having an upregulated target gene. Moreover, for a given status of the target gene, a higher cost is assigned when the control is applied, versus when it is not. In practice, the cost values have to mathematically capture the benefits and costs of intervention and the relative preference of states. They must be set with the help of physicians in accordance with their clinical judgement. Although this is not feasible within the realm of current medical practice, we do believe that such an approach will become feasible when engineering approaches are integrated into translational medicine.

We generate synthetic context-sensitive PBNs in the following manner. Each context-sensitive PBN consists of two contexts. Each constituent network is randomly generated with bias equal to . The bias is the probability that a randomly generated Boolean function takes on a value of one. To complete the specification of a context-sensitive PBN, we need to specify the selection and switching probability distributions, along with its constituent networks. We consider the selection and switching probabilities as parameters during our numerical study. In the first set of experiments, we assume that the constituent networks are selected with equal probabilities. Then, we vary the value of the switching probability. In the second set of simulations, the switching probability is fixed but the selection probability varies. We generate one thousand random synthetic context-sensitive PBNs for each scenario. Our objective is to utilize the statistics generated by these synthetic networks to evaluate the effects of removing the context from the state space of a context-sensitive PBN when designing an intervention strategy.

For each context-sensitive PBN, the exact and approximate transition probability matrices are computed according to (17) and (22), respectively. Thereafter, we solve the optimal intervention problems for the original model and its reduced approximation and find the corresponding optimal strategies.

The devised strategy, , for the exact transition probability matrix specifies the action that should be taken at each time step. The second policy is based on the reduced stochastic matrix and only takes the GAP as its input. Since the performance of the approximate strategy must be evaluated with respect to the dynamics specified by the original model, we need to extend the approximate strategy to elements of . This is achieved by simply disregarding the context element of state  and determining the action based on its GAP element. We denote the resulting intervention strategy obtained through state collapse by .

In the first set of experiments, we determine the effect of the switching probability  on overall performance. To this end, we generate one thousand context-sensitive PBNs for each value of the switching probability. The selection probability is assumed to have uniform distribution for all the generated context-sensitive PBNs. We set the perturbation probability to  for all simulations.

For each context-sensitive PBN generated per the above method, we select a random control gene. Then, we use dynamic programming and derive an optimal intervention strategy  based on the exact transition probability matrix (17). Similarly, an optimal strategy based on the approximate transition probability matrix (22) is derived for the same control gene and is extended to the approximate strategy . For a context-sensitive PBN, we estimate the average total discounted cost induced by the given optimal strategy . To this end, we generate synthetic time-course data for thousand time steps from the transition probability matrix for the context-sensitive PBN, while intervening based on optimal strategy . We estimate the total cost by accumulating the discounted cost of each state given the action at that state. This procedure is repeated ten thousand times for random initial states, and the average of the induced total discounted costs is computed. Following a similar procedure, the approximate strategy  is applied to the system, and the average total discounted cost is computed. Finally, we compute the average total discounted cost for time-course data when no intervention is applied. From here on, we omit the subscript  from the notation of a strategy  to simplify our notations. Since the control gene is selected randomly, this will not affect the following discussions.

The effectiveness of an intervention strategy can be evaluated by computing the difference between its induced cost and the cost accumulated in the absence of intervention. For each set of constituent networks and a given switching probability, we compute the following functions: , , and . These are the average total discounted cost for a given context-sensitive PBN induced by applying optimal strategy , approximate strategy , and no intervention, respectively. The preceding procedure is repeated for one thousand random context-sensitive PBNs, thereby yielding one thousand values for each statistic. We compare the effects of these strategies by computing averages denoted by , , and .

We consider the percentage of reduction in the average total discounted cost as a performance metric. The normalized gain obtained by each intervention strategy is taken as the immediate consequence of the intervention formulation. This metric is defined as the difference between the average discounted cost before and after intervention, normalized by the cost before intervention. The normalized gain corresponding to the optimal strategy  is(28)

and the normalized gain corresponding to the strategy derived from the approximate method  is(29)

**Figure 1:**
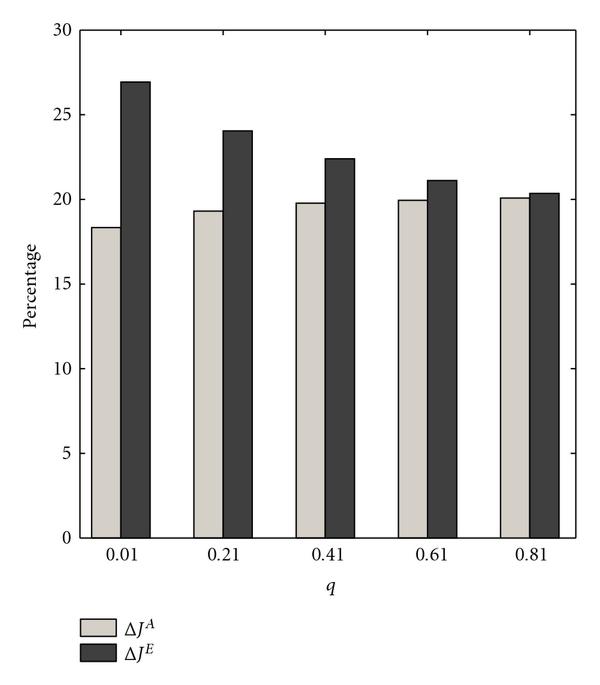
** and  are computed for  context-sensitive PBNs consisting of two contexts**. The switching probability  is the parameter. The selection probability has uniform distribution .

Figure 1 depicts the results of the first experiment, where  is the parameter of interest. As  increases to one, the difference between normalized gains  and  decreases. The approximating method yields close to optimal performance when the switching probability is large, which is outside the range of typical values used for context-sensitive PBNs. If one cannot obtain context knowledge or the number of contexts results in an unacceptable computational burden, the approximate method provides a strategy for the realistic value , which yields a  reduction in performance.

As a byproduct of the intervention formulation, we also consider the effect of an intervention strategy  on the amount of change in the steady-state probability of undesirable states before and after the intervention. For each set of constituent networks and for a given switching probability, we compute  and . These are the normalized reduction in the total probability of visiting undesirable states in the long run for a given context-sensitive PBN when strategies  and  are applied to original system, respectively. In other words, we define(30)

where  is the probability of being in state  in the long run under optimal strategy ;  is the probability of being in state  in the long run under approximate strategy ;  is the probability of being in state  in the long run when no control is applied.

**Figure 2:**
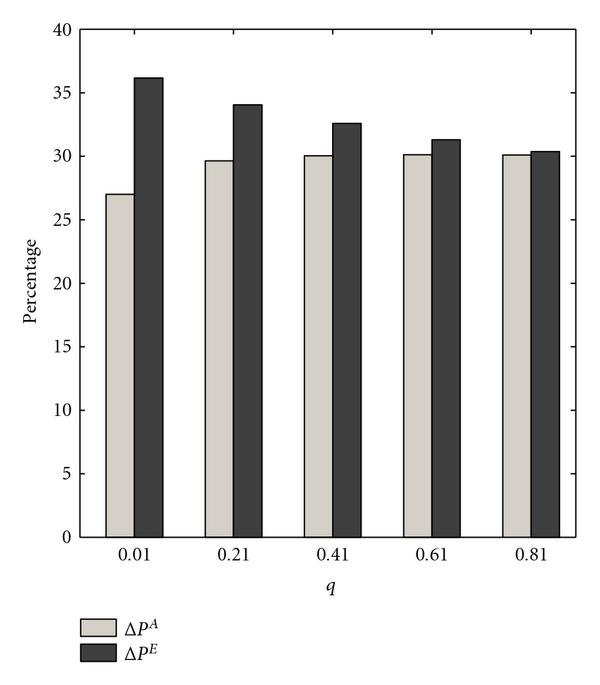
** and  are computed for  context-sensitive PBNs consisting of two contexts**. The switching probability  is the parameter. The selection probability has uniform distribution .

The preceding procedure is repeated for one thousand random context-sensitive PBNs, thereby yielding one thousand values for each statistic. We compare the effect of the strategies devised by the exact and approximate transition probability matrices via the empirical averages of each sample sequence, denoted by  and . Figure 2 shows  and  as functions of the switching probability. The trends are similar to those observed for the normalized gains.

In practice, treatment options, such as chemotherapy, have detrimental side effects. A large number of interventions can cause collateral damage that reduces a patient's quality of life. Thus, we define the quantity  as the expected number of interventions when the strategy  is applied in the long run to gauge these side effects. In particular,  and  are the expected numbers of executed interventions in the long run using the optimal strategy  and the approximate strategy , respectively. We define(31)

where  and  have similar definitions as in (30).

**Figure 3:**
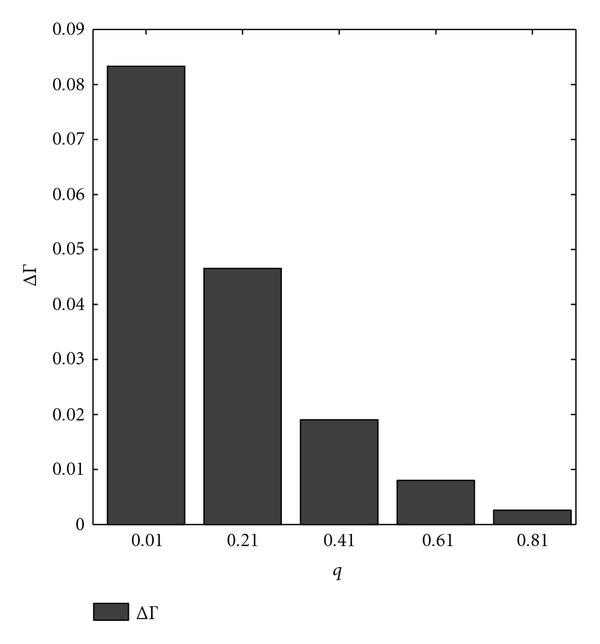
** is computed for  context-sensitive PBNs consisting of two contexts**. The switching probability  is the parameter. The selection probability has uniform distribution .

The preceding procedure is repeated for one thousand random context-sensitive PBNs. We compare the expected number of executed interventions using the difference in empirical averages, denoted by . Figure 3 indicates the variation in  as a function of switching probability . According to this figure, for small switching probabilities, the approximate strategy  is likely to cause more detrimental side effects.

**Figure 4:**
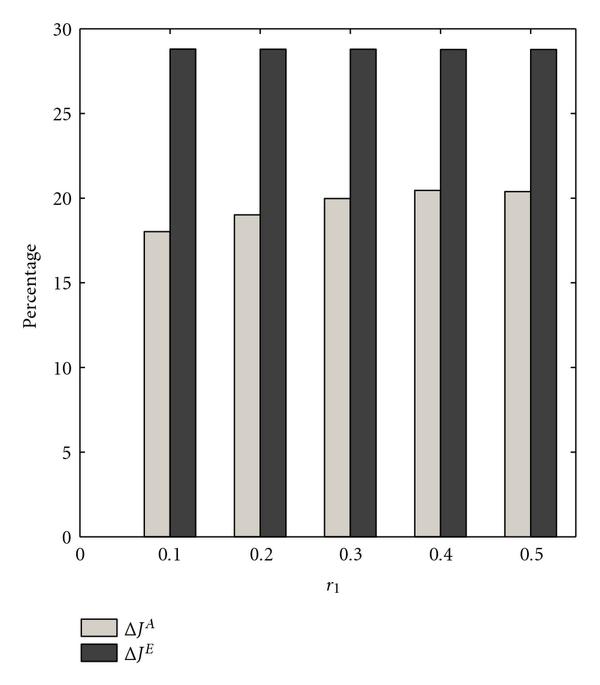
** and  are computed for  context-sensitive PBNs consisting of two contexts**. The switching probability  is . The selection probability of the first constituent network  is varied.

We study the effect of selection probability on the performance of the approximate strategy  in a second set of experiments. We follow the same procedure as before, except that we set , and we vary the probability of selecting each constituent network. We consider two constituent networks so that the selection probabilities are a function of , the probability of selecting the first context. From Figure 4, as  gets smaller, the difference between the performance of strategies  and  diminishes.

**Figure 5:**
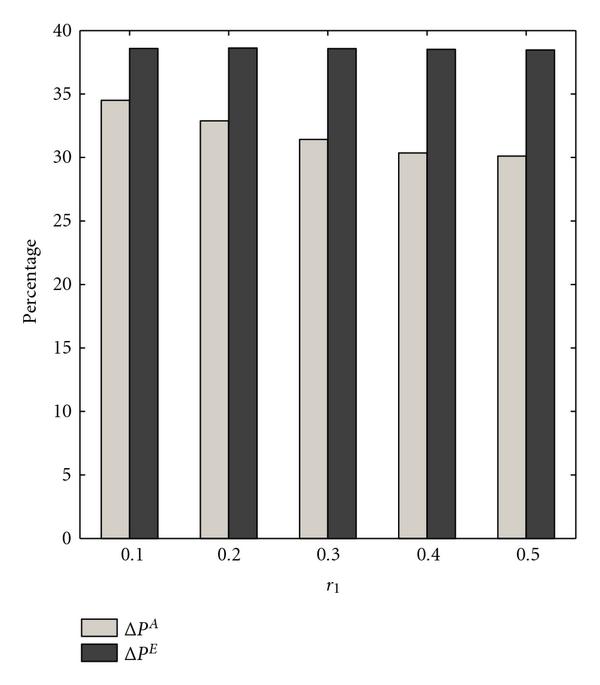
** and  are computed for  context-sensitive PBNs consisting of two contexts**. The switching probability  is . The selection probability of the first constituent network  is varied.

**Figure 6:**
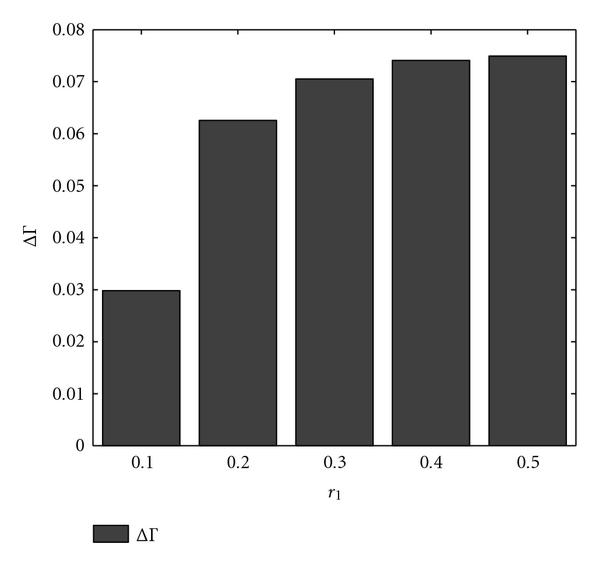
** is computed for  context-sensitive PBNs consisting of two contexts**. The switching probability  is . The selection probability of the first constituent network  is varied.

Figure 5 compares the steady-state measures  and  for the optimal and approximate strategies, respectively. The most interesting observation is that, whereas  decreases as  decreases,  increases. These different behaviors are not contradictory, since the intervention strategy is designed to minimize the total cost and the improvement in the steady-state behavior is a side effect of our goal. We observe that both  and  are stable across parameters, whereas the metrics  and  vary considerably. The context removal approximation affects both  and . That is not the case for the exact transition probability matrix. We suspect that a mathematical analysis of this effect is complicated since it involves interaction between the optimization and the reduction. Finally,  is plotted as a function of the selection probability in Figure 6. Here, we observe that  increases as  decreases.

### 3.2. A Melanoma Case Study

In this section, we compare the performance of the optimal and approximate strategies in the context of a gene regulatory network developed from steady-state data. This steady-state data was collected in a profiling study of metastatic melanoma in which high abundance of messenger RNA for the gene WNT5A was found to be highly discriminating between cells with properties typically associated with high metastatic competence versus those with low metastatic competence [19]. Seven genes were considered in [13, 14]: WNT5A, pirin, S  P, RET , MART , HADHB, and STC . We apply the design procedure proposed in [20] to generate a context-sensitive PBN possessing four constituent networks. The method of [20] generates Boolean networks with given attractor structures, and the overall context-sensitive PBN is designed so that the data points, which are assumed to come from the steady-state distribution of the network, are attractors in the resulting network. The regulatory graphs of these constituent networks can be found in [14]. This approach is reasonable because our interest is in controlling the long-run behavior of the network. The intervention objective for this -gene network is to downregulate WNT5A. The gene WNT5A ceasing to be downregulated is strongly predictive of the onset of metastasis. A number of other intervention studies based on the same data have aimed to downregulate WNT5A. This model has been used since the discovery of the relation between WNT5A and metastasis. The binary nature of the up or down regulation suits our binary model. A state is desirable, that is, belongs to , if WNT5A , and undesirable, that is, belongs to , if WNT5A . As we mentioned earlier, application of intervention requires the designation of desirable and undesirable states, and this depends upon the existence of relevant biological knowledge. The use of WNT5A is one such example where the knowledge of practitioners is incorporated in a theoretical framework. Based on our objective, the cost of control is assumed to be one, and the states are assigned penalties according to the cost-per-stage (27). This is the same cost structure as in [14]. Since our objective is to downregulate WNT5A, a higher penalty is assigned for states having WNT5A upregulated. Also, for a given WNT5A status, a higher penalty is assigned when the control signal is active versus when it is not.

**Figure 7:**
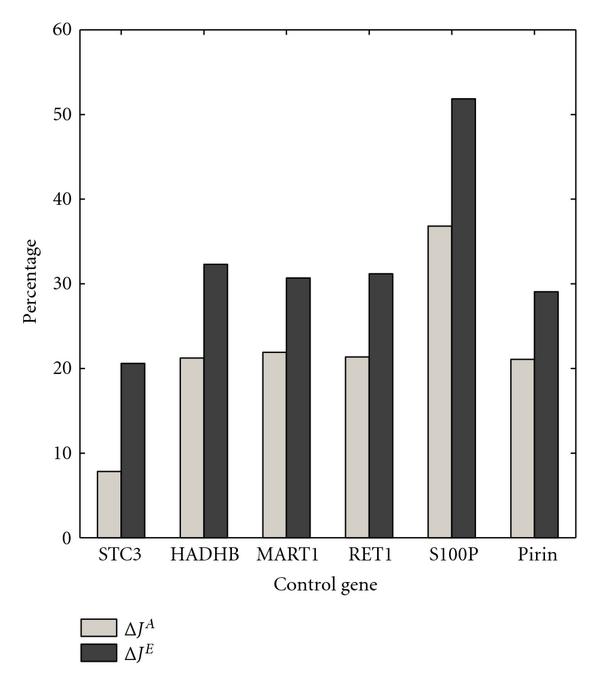
** and  are computed for the WNT5A network for various control genes**. The switching probability is , and the constituent networks are selected with equal probabilities.

The optimal and approximate intervention strategies are found for the melanoma-related context-sensitive PBN when different genes in the network (except WNT5A itself) are employed as the control genes. Figure 7 depicts the normalized gains when the optimal and approximate strategies for each control gene are used to intervene in the context-sensitive PBN. To compute the normalized gains, we computed the costs for ten thousand trajectories of length two hundred thousand. As we expected, the optimal strategy outperforms the approximate strategy significantly for all the control genes. Moreover, for the best control gene S100P, the difference between the two strategies is the greatest.

**Figure 8:**
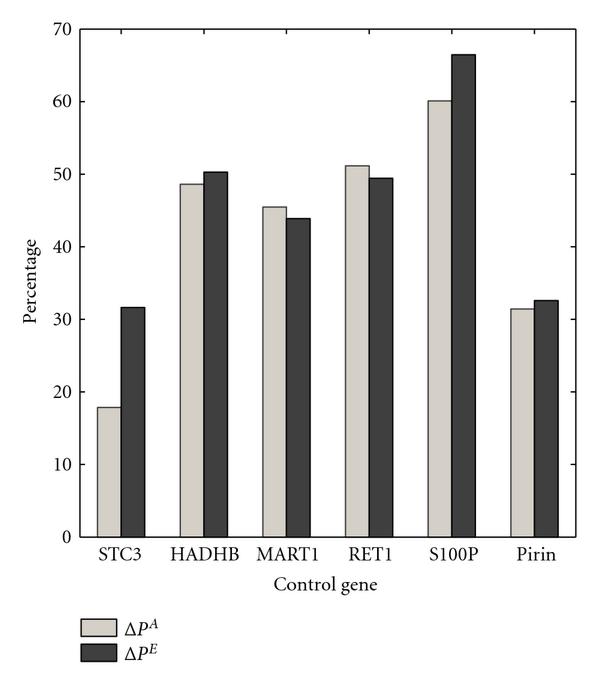
** and  are computed for the WNT5A network for various control genes**. The switching probability is , and the constituent networks are selected with equal probabilities.

Figure 8 depicts the effects of the optimal and approximate strategies on the normalized reduction in the aggregated long-run probability of visiting undesirable states  and , respectively. Here, the strategy based on the S100P outperforms the strategies devised for other control genes. Note that the performance differences are not significant for most of the control genes. In particular, one should not draw any conclusions from the fact that  is slightly less than  in a couple of cases. The intervention strategy is designed to minimize the total cost, and the improvement in the steady-state behavior is a side effect of our method.

**Figure 9:**
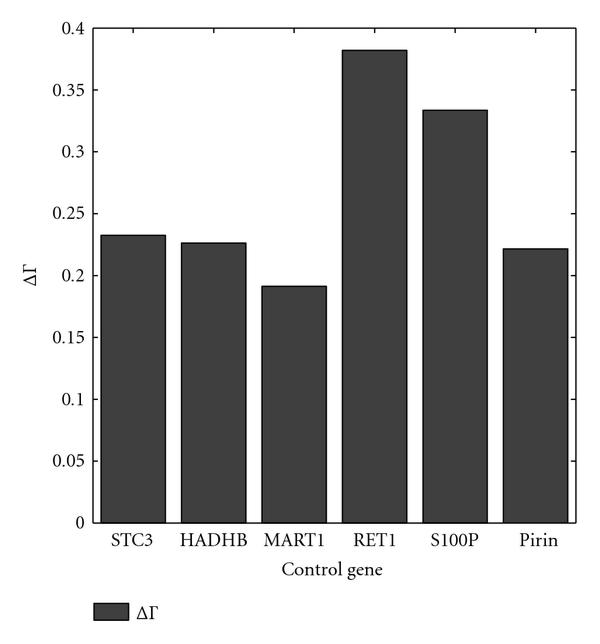
** is computed for the WNT5A network for various control genes**. The switching probability is , and the constituent networks are selected with equal probabilities.

Lastly, Figure 9 shows the difference between the expected number of executed interventions for the optimal strategy and the one derived from the approximate representation of the system. Note that the approximate strategy based on the most effective control gene applies  more interventions compared to the optimal one, while its performance is still worse.

## 4. Conclusion

We have evaluated the effects on intervention performance caused by the proposed reduction in [13, 14] relative to various criteria and values of the parameters of a context-sensitive PBN. We have analytically demonstrated that the reduction method reduces the transition probability matrix of a context-sensitive PBN to the instantaneously random PBN with identical parameters, the fact that is overlooked in [13, 14]. This observation has been demonstrated through extensive numerical studies. We have further studied the relative effectiveness of the devised approximate strategy using several performance criteria: (1) the average normalized gains deduced by the optimal and approximate strategies as indicators of the intervention effectiveness; (2) the normalized reduction in the aggregated probability of visiting undesirable states in the long run as a byproduct of the intervention formulation; (3) the expected number of executed interventions for each strategy. Performance metrics have been compared as functions of both the switching and selection probabilities. In addition, we have compared the optimal and approximate strategies in the framework of a much-studied melanoma-related context-sensitive PBN. The common trend throughout the experiments is that the difference between the performance of the optimal and approximate intervention strategies is small for large switching probabilities. The performance of strategies devised by the reduction method degrades for smaller switching probabilities, which include the range of typical values used for context-sensitive PBNs. It is certainly preferable to design interventions based on the context-sensitive PBN; nevertheless, the approximate model still yields therapeutic benefits in situations where it is impractical to utilize the exact model.

## Appendix

We apply the design procedure proposed in [20] to generate a context-sensitive PBN with four constituent networks. The data used in this inference was collected in a profiling study of metastatic melanoma. To generate the context-sensitive PBN based on the inferred Boolean networks, we set both the switching and perturbation probabilities to . The selection probability distribution is assumed to be uniform . The constituent networks  are reported in Tables 1, 2, 3, and 4, respectively.

Each of Tables 1 to 4 has  rows and  columns, where  denotes the maximum number of predictors for each of the  genes in the network. We set  in this study. The top  rows depict the predictor functions of the genes. We separate the top part of each table from its lower part with a horizontal line to increase the readability. The lower  rows of each table provide the predictors for the genes in the Boolean network. For example, genes , , and  are the predictors of gene  in the constituent network  according to the th row of Table 1. Hence, , the predictor function of gene , can be specified by its  possible outcomes enumerated in the first column of Table 1. Whenever the number of predictors is less than , the outcomes of the predictor function can be enumerated with less than  values. For instance, gene  in Table 1 has two predictors (refer to row  of Table 1), so its predictor function  can be fully specified with  values. According to the the upper part of the third column of Table 1, the value of gene  is set to  when the values of genes  and  are  and , respectively.

**Table 1:** Constituent network

1	1	0	1	0	0	1
1	0	0	0	0	0	0
1	1	1	1	1	0	1
1	1	1	1	1	0	1
1	1		0		1	0
0	0		0		1	1
0	1		0		1	1
0	1		0		1	0
3	5	7				
2	6	1				
3	1					
2	4	7				
3	7					
5	7	1				
3	7	1				

**Table 2:** Constituent network

0	0	0	1	1	0	1
1	0	0	1	0	0	1
0	1	1	1	1	1	0
1	1	1	1	1	1	1
1			0		0	
0			1		1	
0			0		1	
0			1		1	
2	6	1				
2	6					
2	5					
2	4	7				
3	4					
2	5					
5	7					

**Table 3:** Constituent network

1	0	0	0	1	0	1
1	1	0	0	0	0	0
1	1	1	0	0	1	0
0	0	0	0	0	1	1
1		1	1	1	1	
1		0	1	1	1	
0		0	0	1	0	
1		0	1	1	1	
4	5	6				
4	5					
2	4	1				
4	7	1				
3	7	1				
3	5	6				
4	6					

**Table 4:** Constituent network

1	1	0	0	1	1	1
1	0	0	1	0	0	1
1	1	1	1	1	1	0
1	0	0	1	1	1	1
0	1	0		0	0	
0	1	0		0	1	
1	1	0		0	1	
0	1	1		1	1	
2	5	6				
2	4	7				
2	5	7				
2	6					
3	6	7				
3	6	1				
6	7					
